# Engineering controllable CAR T-cell therapies: from binary safety switches to programmable immunity

**DOI:** 10.3389/fimmu.2026.1936293

**Published:** 2026-09-01

**Authors:** Sergei Smirnov, Yuriy Zaritskey, Galina Salogub, Alexander Fonin

**Affiliations:** 1 Group of Bicondensates and Cell Membrane-less Organelles, Institute of Cytology, Russian Academy of Sciences, St. Petersburg, Russia; 2 Laboratory of Genome Editing, Institute of Oncology and Hematology, Almazov National Medical Research Centre, St. Petersburg, Russia

**Keywords:** CAR T-cell therapy, controllable CAR, immunotherapy, logic-gated receptor, modular adaptor, optogenetics, safety switch, synthetic notch

## Abstract

Chimeric antigen receptor (CAR) T-cell therapy has revolutionised cancer gene therapy, yet its expansion into solid tumours is hindered by a critical vulnerability: the autonomous, “always-on” nature of conventional CAR constructs. This unregulated activity drives severe toxicities, including cytokine release syndrome (CRS) and on-target/off-tumour damage, while constitutive signalling in hostile tumour microenvironments (TMEs) accelerates T-cell exhaustion. Early safety strategies relied on irreversible genetic “kill switches,” which sacrifice the therapeutic cell population entirely. This review traces the conceptual evolution of CAR T-cell controllability from binary elimination towards platforms enabling graded, reversible, and spatiotemporally precise regulation. We examine the transition from calibrated signalling architectures and small-molecule-regulated split-CARs to advanced optogenetic and sonogenetic controllers, detailing the biophysics of photoreceptor pairs and their preclinical efficacy. Furthermore, we explore complementary architectures, including autonomous logic-gated receptors. Finally, we propose that the optimal next-generation CAR T product will integrate calibrated signalling, external control, and context-dependent armouring to achieve truly programmable, safe, and durable cellular immunotherapy.

## 1 Introduction: the imperative for controllability in CAR T-cell therapy

Chimeric antigen receptor (CAR) T-cell therapy has established itself as a transformative modality in oncology, achieving unprecedented remission rates in certain hematologic malignancies (1, 2). Second-generation CAR T cells targeting CD19 have achieved complete remission rates of 80–90% in relapsed/refractory B-cell acute lymphoblastic leukaemia (B-ALL) and durable responses in large B-cell lymphoma (3, 4).

Despite these remarkable successes, the broader application of CAR T-cell therapy—particularly in solid tumours and high-burden diseases—remains significantly constrained by a fundamental design flaw: the autonomous, unregulated nature of conventional CAR constructs. Unlike natural T-cell receptors, which are tightly regulated by complex checkpoints and co-stimulatory requirements, conventional CARs are constitutively active. This “always-on” architecture drives three interconnected clinical challenges: (1) severe acute toxicities including CRS and ICANS (5, 6); (2) on-target/off-tumour toxicity, as exemplified by fatal pulmonary toxicity following HER2-targeted CAR T-cell administration (7); and (3) T-cell exhaustion and loss of persistence, characterised by upregulation of TOX and NR4A transcription factors and multiple inhibitory receptors (PD-1, TIM-3, LAG-3) (8–11).

Existing reviews on controllable CAR-T therapies often describe technologies chronologically or by molecular mechanism, without providing a framework that directly informs clinical decision-making (1–3). Our Tier 1–3 classification is designed to be clinically intuitive: it organises the field by the level of control each platform provides (from irreversible elimination to reversible modulation to spatiotemporal precision) and by its clinical maturity. This framework helps researchers and clinicians understand which platforms are currently viable for clinical translation and which remain at the preclinical stage. By organising the field according to clinical maturity and practical applicability rather than purely technological novelty, our classification advances the current understanding by providing an actionable roadmap for platform selection in clinical trial design.

## 2 Tier 1 control: irreversible elimination systems (“emergency brakes”)

The initial phase of engineering controllability into CAR T-cell therapies was driven by an urgent need to manage severe, life-threatening toxicities (12). These “kill switch” systems represent the first generation of control mechanisms, designed to provide a fail-safe mechanism for the complete and rapid elimination of the engineered cell population in the case of catastrophic adverse events (13). This approach fundamentally addresses patient safety at the expense of therapeutic persistence, functioning as an irreversible “off switch”.

**Figure 1:**
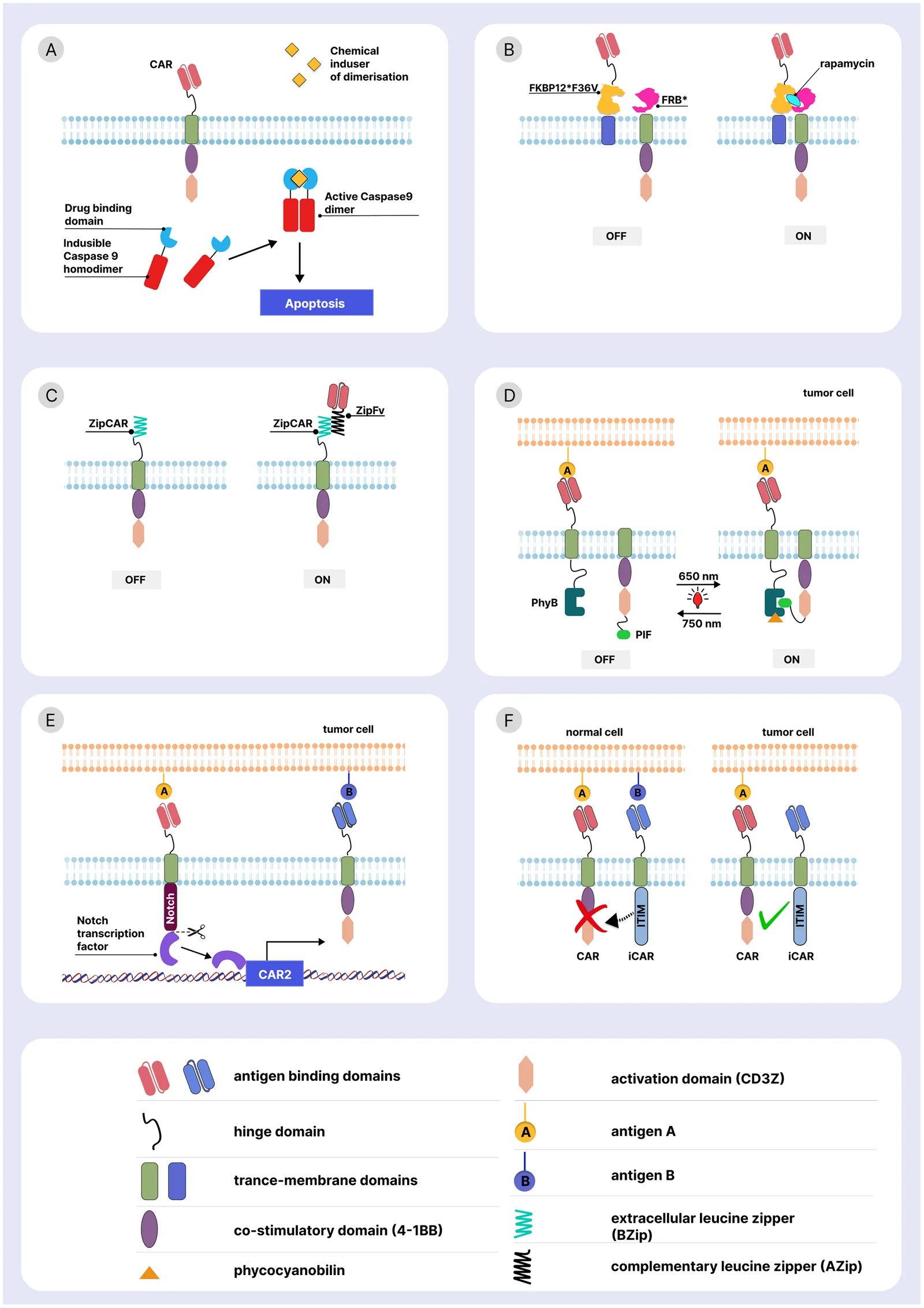
Evolution of controllable CAR T-cell systems. **(A)** Phase 1: Irreversible elimination (iCasp9). The CAR T cell expresses a modified inducible caspase-9 (iCasp9) fused to a drug-binding domain. Administration of a synthetic homodimeriser (e.g., AP1903) forces proximity and dimerisation of iCasp9 molecules, triggering a rapid caspase cascade and apoptosis of the therapeutic cells within minutes, serving as an emergency safety brake. **(B)** Phase 2: Reversible pharmacological control (split-CAR). Split-CAR systems (e.g., DARIC platform) employ extracellular antigen-binding and intracellular signalling domains expressed as separate polypeptides, each fused to mutated dimerisation domains (FRB* and FKBP12*F36V). The addition of a small-molecule bridge (e.g., rapamycin or its non-immunosuppressive analogues) induces heterodimerisation, assembling a functional CAR receptor. **(C)** Phase 2: Modular adaptor platforms (SUPRA CAR). The SUPRA CAR platform separates the CAR into a universal receptor (zipCAR) on the T cell and a target-specific soluble adapter (zipFv). The zipCAR displays an extracellular leucine zipper (BZip), while the zipFv comprises a targeting scFv fused to a complementary leucine zipper (AZip). Upon co-administration, the two leucine zippers heterodimerise, forming a functional CAR complex that redirects T-cell cytotoxicity towards tumour cells expressing the antigen recognised by the zipFv. By switching the scFv in the adapter, the same universal T cell can be redirected against different tumour-associated antigens, providing flexibility to address antigen heterogeneity. **(D)** Phase 3: Spatiotemporally precise optogenetic control (PhyB-PIF6). The PhyB-PIF6 system utilises phytochrome B (PhyB) containing a covalently bound linear tetrapyrrole chromophore (e.g., phycocyanobilin, PCB). Upon illumination with red light (~650 nm), the chromophore undergoes photoisomerisation from the C15-Z to the C15-E configuration, triggering a conformational rearrangement that exposes a hydrophobic pocket binding phytochrome-interacting factor 6 (PIF6) with high affinity, thereby assembling the active CAR complex. Illumination with far-red light (~750 nm) reverses this isomerisation, actively dissociating the complex and providing a rapid “off-switch”. **(E)** Complementary architecture: AND-gate logic (SynNotch). A synthetic Notch (synNotch) receptor recognises a primary tumour antigen (Antigen A) on the cell surface, triggering proteolytic cleavage and nuclear translocation of its intracellular domain. This drives transcription of a secondary CAR targeting Antigen B, ensuring cytotoxic activity is unleashed only against double-positive tumour cells, thereby sparing healthy tissues expressing only one antigen. **(F)** Complementary architecture: NOT-gate logic (inhibitory CARs and Tmod). Inhibitory CARs (iCARs) incorporate an extracellular domain recognising a healthy-tissue antigen coupled to an intracellular inhibitory ITIM (from PD-1 or CTLA-4). When the T cell encounters both tumour and healthy antigens, the inhibitory signal overrides activation, protecting normal tissues.

The iCasp9 system (**Figure 1A**) is the most clinically advanced kill switch. The clinical value of this system has been demonstrated in multiple early-phase trials. In a pivotal phase I study of P-PSMA-101 for metastatic castration-resistant prostate cancer, eight patients experienced severe toxicity necessitating activation of the integrated iCasp9 switch; in seven of these eight cases, the severe toxicities (including CRS and ICANS) were successfully resolved following switch activation (14). Similar safety switches have been incorporated into B7-H3-targeted CAR-T cells for pancreatic cancer (NCT06158139) (15) and GD2-targeted CAR-T cells for neuroblastoma (NCT03721068) (16), demonstrating the broad applicability of this approach across solid tumour indications.

The fundamental trade-off of Tier 1 systems remains: safety is achieved by sacrificing the therapeutic cell population, eradicating the very agents needed for sustained anti-tumour immunity. Therefore, while indispensable for patient safety, irreversible switches do not permit graded modulation of the immune response.

## 3 Tier 2 control: reversible pharmacological modulation platforms

### 3.1 Split-CAR systems and conditional dimerisation

A key step beyond irreversible kill switches is the development of pharmacological “dimmer switches” that allow reversible modulation of CAR T-cell activity without eliminating the cell population. Split-CAR systems embody this concept by physically uncoupling antigen recognition from signalling domains, enabling tunable and reversible control (17). The DARIC platform (**Figure 1B**) operationalises this principle via rapamycin-dependent dimerisation (18). However, clinical translation of this elegant design has proven challenging. The SC-DARIC33 programme for CD33+ relapsed/refractory acute myeloid leukaemia entered a phase I trial (PLAT-08, NCT05105251) but was placed on clinical hold by the FDA in 2023 following a fatal adverse event, and as of mid-2026 remains stalled.

Several key obstacles have hindered the clinical translation of split-CAR systems. First, pharmacokinetic complexity of the dimeriser: rapamycin analogues require chronic dosing and exhibit variable bioavailability, making it difficult to maintain the precise plasma concentrations needed for optimal CAR assembly without causing off-target mTOR inhibition (17). Second, manufacturing complexity: split-CAR constructs require the coordinated expression of two separate polypeptide chains at balanced stoichiometric ratios, which is significantly more challenging to achieve consistently under GMP conditions compared to single-chain CAR designs. Third, the clinical burden of continuous dimeriser administration adds substantial logistical complexity, as patients must adhere to strict dosing schedules to maintain CAR activity, unlike standard CAR-T products that function autonomously after infusion. These challenges highlight the substantial gap between preclinical proof-of-concept and human safety for split-receptor systems, and have shifted clinical focus toward modular adaptor platforms.

### 3.2 Modular adaptor platforms

A more clinically advanced form of reversible control is the modular adaptor system. In this architecture, the CAR T cell is rendered inert or “universal”, capable of recognising a universal tag, while a separate, short half-life soluble adaptor molecule links the T cell to the tumour antigen (19). By controlling the administration and concentration of the adaptor, clinicians can precisely turn the therapy on, off, or dial down its intensity. Unlike split-CAR systems that require chronic oral dosing of small-molecule dimerisers with long half-lives and variable bioavailability, modular adaptor platforms use short-lived protein-based adaptors administered as titratable intravenous infusions that can be rapidly discontinued, enabling deactivation within hours rather than days.

The UniCAR platform utilises a universal CAR T cell combined with a targeting module (TM). In a phase I dose-escalation study of UniCAR-T-CD123 for R/R AML, the platform demonstrated robust clinical activity and proof-of-concept for this modular approach. Among heavily pre-treated patients, the overall response rate reached 56%, with 31% achieving complete remission (CR/CRi), and all evaluable MRD-positive patients converted to MRD-negative status (20, 21). The safety profile was favourable, with CRS mostly grade 1–2 and only one grade 3 event. Crucially, when grade 3 CRS or grade 2 ICANS occurred, toxicities resolved within 24 hours after stopping the TM infusion, with rapid recovery of haematopoietic parameters (21). Interruption and re-administration of the TM in consolidation cycles resulted in robust re-expansion of UniCAR-T cells and deeper remissions, demonstrating that the system can be turned “off” and “on” again as needed (21).

The SUPRA CAR platform (**Figure 1C**) separates the CAR into a universal T-cell receptor and a target-specific adaptor (22). In a phase I trial of CLBR001 + SWI019 (anti-CD19 switch) for heavily pre-treated R/R B-cell malignancies (NCT04450069), the combination achieved an overall response rate of 78% and a complete response rate of 67% among the first 9 evaluable patients (22). The switchable design enabled precise pharmacological control: withholding or reducing SWI019 dosing shortened the duration of CRS/ICANS events to 2–3 days *versus* 5–17 days with conventional CAR-T products. CLBR001 cells engrafted at higher levels in peripheral blood over the first 90 days compared with commercial CAR-T products. No dose-limiting toxicities were reported, and clinical activity was only observed following SWI019 administration, demonstrating that the SUPRA platform provides robust efficacy with a favourable safety profile (22).

## 4 Tier 3 control: physical and biological precision systems

The third tier of CAR T-cell control represents the frontier of bioengineering, moving beyond systemic pharmacological regulation to achieve precise spatiotemporal control using physical stimuli. These advanced systems aim to overcome the inherent limitations of small molecules—systemic distribution, pharmacokinetics, and poor tissue penetration—by harnessing external physical energy sources like light or ultrasound.

### 4.1 Optogenetics: light-controlled CAR assembly

Optogenetics is the most extensively developed paradigm within this tier, exploiting light-induced allosteric regulation to control CAR assembly. This approach allows clinicians to turn CAR T-cell activity on and off with precise pulses of light (23).

Blue-light systems (e.g., CRY2-CIB1 and LOV domains) utilise flavin-based chromophores naturally present in mammalian cells. However, dissociation in darkness is relatively slow (4–5 minutes for CRY2-CIB1), which can prolong signalling beyond the intended pulse (24, 25). Faster off-kinetics are achieved with LOV-based systems (e.g., TULIP and other LOV-domain platforms), enabling tighter temporal control at the cost of lower quantum yield—the fraction of absorbed photons that trigger the desired conformational change. A lower quantum yield demands higher light intensity or longer exposure to achieve effective activation, which may limit tissue penetration depth and increase phototoxicity risks (26, 27).

Despite these attractive features, blue-light systems suffer from poor tissue penetration, limiting their applicability for most solid tumours (28). Nano-optogenetics offers a workaround using upconversion nanoparticles (UCNPs) to convert deeply penetrating near-infrared light into visible blue light, activating optogenetic modules precisely at the tumour site (27). However, nanoparticle delivery and potential long-term toxicity remain significant translational hurdles.

Red and far-red light systems based on phytochromes (e.g., PhyB-PIF6 system, **Figure 1D**) can penetrate several centimetres through biological tissues, raising the possibility of non-invasive control in deep-seated tumours (29). However, red/far-red systems carry substantial limitations preventing clinical translation: exogenous chromophore dependency (mammalian cells do not synthesise bilins), high immunogenicity of plant-derived PhyB/PIF6 proteins, large genetic size exceeding lentiviral packaging limits, and regulatory complexity as “combination products” (cell therapy plus light-delivery device) (24).

### 4.2 Sonogenetics: ultrasound-induced control

Sonogenetics has emerged as a highly promising alternative for deep tissues, offering deep tissue penetration (up to 10–15 cm), millimetre spatial resolution, and clinical ubiquity (ultrasound is already widely used in diagnostic imaging and thermal ablation) (30). The EchoBack-CAR platform represents the most advanced sonogenetic system for CAR T-cell control. This platform employs a heat-inducible promoter (HSP70) activated by focused ultrasound (FUS)-induced hyperthermia (42–45 °C), triggering CAR expression exclusively within the irradiated region. A self-amplifying feedback architecture reinforces CAR expression upon antigen encounter, creating a self-sustaining transcriptional programme within the TME. When T cells migrate away from the ultrasound focus or antigen is cleared, CAR expression wanes, minimising off-target injury (30).

Sonogenetics offers distinct advantages over optogenetic approaches: no exogenous chromophore requirement, deep tissue penetration, non-immunogenic components (human heat-shock proteins), and clinically available hardware. However, translational barriers remain: need for specialised ultrasound hardware with real-time temperature monitoring, potential HSP70 promoter leakiness, thermal safety concerns, and lag time (hours before CAR protein is synthesised). The dual-control mechanism—external physical trigger plus endogenous antigen feedback—provides unprecedented spatial and temporal precision, positioning sonogenetics as a promising candidate for future clinical translation despite these challenges (30).

### 4.3 Genetic code expansion: translational control

Genetic code expansion (GCE) represents a fundamentally distinct approach from Tier 2 pharmacological modulation: rather than controlling the activity of pre-existing CAR molecules, GCE regulates CAR synthesis at the translational level itself (31). This approach employs an orthogonal tRNA/aminoacyl-tRNA synthetase pair that incorporates a non-canonical amino acid (ncAA) at a specific amber stop codon (UAG) engineered into the CAR sequence. In the absence of the ncAA, translation stalls, producing a truncated, non-functional receptor fragment. Only when the ncAA is supplied exogenously (via oral or intravenous administration) is full-length CAR protein synthesised and inserted into the T-cell membrane. This provides a stringent “on/off” switch at the level of protein biogenesis itself, with the advantage that no exogenous chromophores or light-delivery hardware are required. However, significant barriers remain: the need for stable delivery of orthogonal machinery (which may itself be immunogenic), the pharmacokinetics and bioavailability of ncAAs in vivo, and the potential for residual “leaky” translation in the absence of the ncAA (31).

## 5 Complementary strategies: logic-gating, microenvironmental remodelling and emerging technologies

### 5.1 Autonomous logic-gating and tumour context recognition

Beyond external control, parallel innovation focuses on endowing CAR T cells with autonomous sensing capabilities. **Figure 1E** illustrates the synthetic Notch (synNotch) receptor implementing AND-gate logic. A synNotch receptor recognises a primary tumour antigen (Antigen A), triggering proteolytic cleavage and nuclear translocation of its intracellular domain, which drives transcription of a secondary CAR targeting Antigen B. Cytotoxic activity is unleashed only against double-positive tumour cells (32, 33). The E-SYNC programme is the first-in-human trial applying this to EGFRvIII-positive glioblastoma (NCT06186401). In the initial dose-escalation cohort, autologous E-SYNC T-cells were successfully manufactured for all nine patients, and eight patients have been treated (six at dose level 1 and two at dose level 2), with no serious adverse events or dose-limiting toxicities observed (34). Encouragingly, two of six patients at the lowest dose level remain progression-free at 20 and 15 months following initial resection. However, in two patients who recurred within two months of infusion, analysis of recurrent tumour tissues showed loss of EGFRvIII despite infiltration of E-SYNC cells, highlighting a potential resistance mechanism. These early data demonstrate that the synNotch-based “prime-and-kill” approach is feasible and safe in this poor-prognosis patient population, with promising signals of prolonged progression-free survival (34).

Inhibitory CARs (iCARs) provide NOT-gate logic by coupling an extracellular domain specific for a healthy tissue antigen to an intracellular inhibitory motif (**Figure 1F**) (35). When a T-cell engages both tumour and healthy tissue antigens, the inhibitory signal overrides activation. However, this design faces two fundamental limitations. First, incomplete inhibition occurs when normal tissues express target antigens at densities insufficient to cluster Immunoreceptor Tyrosine-based Inhibitor Motifs (ITIMs), allowing strong activating signals from conventional CARs to prevail (36). Second, tonic signalling arises from spontaneous scFv clustering in the absence of antigen—particularly for scFvs bearing positively charged surface residues—leading to constitutive ITIM activation that may drive T-cell exhaustion, receptor desensitisation, or paradoxical activation via compensatory signalling pathways (37). These challenges have motivated more sophisticated NOT-gate architectures, such as the Tmod platform, which exploits tumour-specific HLA loss of heterozygosity rather than relying on differential antigen density.

The Tmod platform (A2B694) implements NOT-gate logic to kill mesothelin-positive tumour cells that have lost HLA-A02 expression, sparing normal tissues. Preclinical validation demonstrated potent antitumor activity against HLA-A02-negative tumour cells while preserving healthy tissues (38). In the ongoing phase 1/2 EVEREST-2 trial (NCT06051695), as of May 2025, seven patients with advanced solid tumours (ovarian, pancreatic, NSCLC, and colorectal) had been treated (39). A2B694 demonstrated manageable safety and tolerability with no dose-limiting toxicities reported (39). Notably, one patient with KRASG12V/STK11 co-mutated non-small cell lung cancer achieved a complete response at day 90 post-infusion, confirmed at day 180—the first reported complete response to a CAR-T cell therapy in NSCLC (39, 40). However, the Tmod approach is constrained by the relatively narrow patient population eligible for such treatment, as HLA-A*02 loss of heterozygosity occurs in approximately 20–30% of colorectal, pancreatic, and NSCLC cases (41).

### 5.2 Emerging complementary approaches

Beyond logic-gating, several emerging strategies are reshaping the CAR-T engineering landscape without fitting neatly into the Tier 1–3 controllability framework.

Cytokine-armoured CAR-T cells (TRUCKs) are engineered to secrete immunomodulatory payloads upon activation, remodelling the immunosuppressive tumour microenvironment to prevent exhaustion (42). The most clinically advanced example is the IL-7/CCL19-armoured CD19 CAR-T (7×19 CAR-T). In an extended 5-year follow-up of a phase I trial (NCT04833504) involving 39 adults with relapsed/refractory large B-cell lymphoma (41% with ECOG PS 2–3), 7×19 CAR-T demonstrated sustained efficacy: 28.2% of patients maintained ongoing responses at a median follow-up of 63 months, with 5-year overall survival of 43.6% (43). For comparison, the 5-year overall survival in the pivotal JULIET trial of tisagenlecleucel (Kymriah) was 31.7%. No late-onset severe toxicities emerged, and single-cell analyses identified FOXO1^+^THEMIS^+^ memory T cells as a biomarker of long-term remission, constituting 5.1% of CAR-T cells in complete responders—a 3.6- to 5.7-fold increase compared to relapsed or non-responder groups (43).

Another armoured approach, huCART19-IL18 (NCT04684563), combines a CD19-targeting CAR with inducible IL-18 secretion. In a first-in-human trial of 21 patients with lymphoma who had failed prior anti-CD19 CAR-T therapy, the treatment demonstrated a manageable safety profile (62% CRS, mostly mild-to-moderate; 14% mild neurotoxicity) and achieved an 81% overall response rate at 3 months, with 52% complete remissions (44).

For solid tumours, C-CAR031 (AZD7003)—a GPC3-targeted CAR-T therapy armoured with a dominant-negative TGFβ receptor II (dnTGFβRII)—has demonstrated encouraging activity in a phase I trial (NCT06590246) involving 36 heavily pre-treated patients with advanced hepatocellular carcinoma (median four prior lines) (45). Tumour regression occurred in 32 of 36 patients (88.9%), with a median best reduction of 41.6% (range: 3.4–94.4%). The objective response rate was 44.4% (all partial responses), with a median duration of response of 4.4 months. Median progression-free survival was 4.2 months, and median overall survival reached 14.2 months (45). CRS was mostly grade 1–2 (only 2 cases grade 3), with no dose-limiting toxicities or ICANS. These data provide the first clinical evidence that dnTGFβRII armouring can counteract TGFβ-mediated suppression and enable meaningful antitumour activity in an immunosuppressive solid tumour indication.

In contrast to cytokine armouring, which addresses environmental factors limiting CAR-T persistence, CRISPR-based engineering enables precise genomic integration at safe harbour loci, potentially reducing manufacturing variability and improving persistence (46). The most mature clinical data come from BRL-201, a non-viral CRISPR/Cas9-mediated anti-CD19 CAR-T with PD-1 locus-specific integration. In a first-in-human phase I trial (NCT04213469) with 4-year follow-up, BRL-201 demonstrated durable remissions in patients with relapsed/refractory non-Hodgkin lymphoma, with no off-target CRISPR effects detected (47).

Additional CRISPR-edited allogeneic platforms are advancing clinically. CB-010, an allogeneic anti-CD19 CAR-T, is being evaluated in a phase I trial (NCT06752876) for refractory systemic lupus erythematosus, representing the first application of CRISPR-edited CAR-T cells in autoimmune disease. CTX130, a CD70-targeted allogeneic CRISPR-Cas9-engineered CAR-T (NCT04502446), has shown manageable safety and preliminary activity in relapsed/refractory T-cell malignancies (48). CRISPR Therapeutics is also advancing CTX-112 (NCT05643742) and CTX-131 (NCT05795595) targeting B-cell malignancies and solid tumours, with additional gene edits (e.g., Regnase-1, TGFBR2) designed to enhance CAR-T fitness and overcome immunosuppression.

In parallel with genetic and pharmacological approaches, metabolic reprogramming has emerged as a critical strategy to counteract T-cell exhaustion and enhance persistence within the hostile tumour microenvironment. Conventional CAR-T cells undergo a metabolic shift from oxidative phosphorylation to aerobic glycolysis upon activation, a state that accelerates terminal differentiation and exhaustion. Engineering CAR-T cells to favour fatty acid oxidation and mitochondrial fitness—for instance, through overexpression of the transcriptional co-activator PGC-1α, which promotes mitochondrial biogenesis, or via pharmacological activation of the PGC-1α pathway—has been shown to preserve stem-cell memory phenotypes and improve long-term anti-tumour efficacy in preclinical models (49, 50). Notably, a clinically approved PGC-1α agonist, bezafibrate, has demonstrated the ability to enhance CAR-T cell metabolic activity and therapeutic outcomes when used during ex vivo manufacturing (50). When combined with controllable platforms, metabolic armouring ensures that engineered T cells maintain the functional fitness required for durable, programmable responses.

Concurrently, the integration of artificial intelligence (AI) and machine learning is revolutionising the design phase of CAR-T therapies. AI-driven models are increasingly utilised to predict and optimise the binding affinity and specificity of single-chain variable fragments, reduce their immunogenicity, and design optimal linker regions to minimise tonic signalling (51, 52). Deep learning algorithms are being deployed to predict epitope-paratope interactions and engineer novel synthetic receptors or logic-gated circuits with enhanced precision, significantly accelerating the transition from empirical trial-and-error to rational, computationally guided receptor design (52).

A detailed discussion of these approaches lies beyond the scope of this mini-review, but their integration with controllable platforms represents a promising frontier for next-generation CAR-T therapies.

## 6 Clinical decision-making and future perspectives

### 6.1 Clinical decision-making framework

The choice of a controllable CAR-T platform must be guided by the dominant clinical risk in each disease context. Rather than treating all platforms as interchangeable, the decision should be driven by the specific challenge—whether it is acute toxicity, narrow therapeutic windows, or tumour heterogeneity.

For high-tumour burden B-cell malignancies, where life-threatening CRS and neurotoxicity driven by massive, synchronous T-cell activation are the primary concerns, reversible pharmacological control offers the optimal solution. Among the available options, modular adaptor platforms (UniCAR, SUPRA CAR) have demonstrated clear clinical advantages over split-CAR systems. Split-CARs (DARIC) require chronic oral dosing of a small-molecule dimeriser with variable bioavailability and carry the risk of immunosuppressive mTOR inhibition, factors that contributed to a clinical hold (17). In contrast, modular adaptors use short-lived intravenous adaptors that can be rapidly discontinued, enabling de-escalation within hours rather than days (19). This preserves the therapeutic cell population for sustained remission while providing an immediate safety valve for acute toxicities, a critical advantage over irreversible kill switches which permanently eliminate the cells needed for durable disease control.

Acute myeloid leukaemia presents a more complex challenge due to the narrow therapeutic window imposed by target antigen expression on healthy haematopoietic stem cells. Here, modular adaptors provide the essential safety mechanism to permit marrow recovery during cytopenias (20, 21), while also enabling sequential or simultaneous targeting of multiple antigens through adaptor switching—a critical advantage in a disease characterised by antigen heterogeneity. NOT-gate architectures, such as the Tmod platform, offer a complementary strategy by enhancing tumour selectivity through inhibition upon recognition of healthy-tissue antigens—for example, pairing a CD33-directed activator with a CD16b-directed blocker to protect normal myeloid cells (38).

For solid tumours with shared antigens—where on-target/off-tumour toxicity and antigen escape represent the principal barriers—autonomous logic-gating offers the most clinically robust approach. AND-gate systems (synNotch) ensure cytotoxic activity is triggered exclusively upon recognition of a tumour-specific priming antigen, whereas NOT-gate designs (Tmod) protect normal tissues by actively suppressing activation in the presence of healthy-cell markers (34, 39). The Tmod platform has already demonstrated the first complete response to CAR-T therapy in non-small cell lung cancer (39, 40), validating the clinical potential of this approach. For solid tumours, biological control systems confer spatial and molecular precision unattainable through pharmacological modulation alone.

Overall, the selection of a controllable CAR-T platform should not be driven by technological novelty alone but by the specific risk-benefit profile best suited to each clinical scenario. The Tier 1–3 framework provides a structured approach to this decision-making, mapping the level of control to the dominant clinical challenge (**Table 1**).

**Table 1:** Landscape of controllable CAR T-cell platforms.

Control tier & platform	Core mechanism & design	Clinical status	Comparative advantages & clinical utility	Critical translational, GMP & regulatory barriers
Tier 1: Irreversible Elimination (iCasp9)	Dimeriser-induced caspase cascade triggers apoptosis of the entire T-cell population.	Phase I (prostate, pancreatic, neuroblastoma)	Simplest design: Lowest GMP manufacturing complexity and production cost among controllable platforms. Proven clinical safety for acute rescue.	Irreversible:Unlike Tiers 2/3, it sacrifices the therapeutic cells, requiring costly re-infusion if disease recurs. No graded modulation.
Tier 2: Split-CAR (DARIC)	Rapamycin-induced heterodimerisation of physically separated CAR recognition and signalling domains.	Phase I (AML; clinical hold 2023)	Reversible without cell loss:Preserves T-cell population (unlike Tier 1). Allows dose-dependent tuning.	High GMP complexity: Requires coordinated expression of two separate chains at strict stoichiometric ratios. Chronic oral dosing burden; risk of mTOR-mediated immunosuppression.
Tier 2: Modular Adaptor (UniCAR, SUPRA CAR)	Universal CAR T-cell recognises a short half-life, target-specific soluble adaptor protein.	Phase I/II (AML, B-cell malignancies)	Rapid titration: Deactivation within hours (faster than split-CARs). Switchable targets allow adaptation to antigen heterogeneity without re-manufacturing T-cells.	Logistical burden: Requires continuous/frequent IV adaptor infusions. Adaptor manufacturing adds to overall cost. Potential adaptor immunogenicity limits long-term scalability.
Tier 3: Optogenetics (PhyB-PIF6, CRY2-CIB1)	Light-induced conformational change reassembles split CAR domains with precise spatiotemporal control.	Preclinical	Unmatched precision: Sub-millimetre spatial and second-scale temporal control. Non-invasive (blue/red light) compared to physical implants.	Vector size limits: Genetic payload exceeds standard lentiviral capacity (~8-9 kb). PhyB requires exogenous chromophores; CRY2 has poor tissue penetration. High regulatory complexity as a "combination product" (cell + light device).
Tier 3: Sonogenetics (EchoBack-CAR)	Focused ultrasound-induced local hyperthermia activates a heat-shock promoter (HSP70) driving CAR expression.	Preclinical	Deep tissue reach: Penetrates 10-15 cm without exogenous chromophores. Utilises clinically ubiquitous ultrasound hardware (unlike specialised optogenetic lasers).	Hardware dependency: Requires specialised FUS hardware with real-time temperature monitoring. Thermal safety risks (42-45°C). Lag time of hours for protein synthesis vs. seconds for optogenetics.
Autonomous Logic: AND-gate (SynNotch)	Primary antigen recognition drives the de novo transcription of a secondary CAR targeting a second antigen.	Phase I (glioblastoma)	Autonomous spatial precision: Requires no external triggers or chronic dosing (unlike Tiers 2/3). Highly specific "prime-and-kill" mechanism.	Massive genetic payload:Exceeds single lentiviral capacity, requiring dual-vector or transposon systems, severely complicating GMP manufacturing and increasing cost. High risk of antigen escape.
Autonomous Logic: NOT-gate (iCARs, Tmod)	Inhibitory motifs (ITIMs) triggered by healthy-tissue antigens (iCAR) or tumour-specific HLA loss (Tmod) override activation.	Preclinical / Phase I/II (Tmod)	Active protection: Suppresses activation upon encountering healthy tissues, addressing on-target/off-tumour toxicity more robustly than simple antigen-density tuning.	Signal balancing: iCARs suffer from tonic signalling and incomplete inhibition at low antigen densities. Tmod is restricted to a narrow patient subset (~20-30%) with HLA loss of heterozygosity.

### 6.2 Translational barriers

Beyond biological considerations, several practical barriers impede the clinical translation of controllable CAR-T platforms. Vector size limitations pose a significant challenge for complex constructs: the PhyB-PIF6 optogenetic system, for example, exceeds lentiviral packaging limits (~8–9 kb), necessitating alternative delivery strategies such as transposon-based systems, dual-vector approaches, or non-viral delivery platforms (53). Logic-gated receptors such as synNotch also require large genetic payloads, complicating vector design and manufacturing (54).

Manufacturing complexity and cost remain prohibitive for widespread adoption. The production of autologous CAR-T cells is intrinsically complex, time-consuming, and costly, with vein-to-vein workflows requiring apheresis, cryopreservation, cold-chain logistics, and weeks of ex vivo transduction (53, 54). Split-CAR constructs, which require the coordinated expression of two separate polypeptide chains at balanced stoichiometric ratios, add an additional layer of manufacturing difficulty compared to single-chain CAR designs (17).

Regulatory complexity is amplified for combination products that pair cell therapy with a pharmacological agent (e.g., rapamycin for DARIC or adaptor molecules for modular platforms) or a medical device (e.g., light-delivery systems for optogenetics). Combination products are defined by the FDA as products combining drugs, devices, and/or biologics, and their component parts may be subject to different regulatory pathways, requiring coordination between multiple centres (e.g., CBER, CDER, CDRH) (55). These products require coordinated approval from multiple regulatory bodies and present challenges for standardised quality control. Scalability is another concern: modular adaptor platforms require continuous or frequent adaptor administration (19), adding logistical complexity and cost to treatment regimens, while the bespoke, patient-specific nature of autologous CAR-T manufacturing precludes the scalable production achievable with conventional pharmaceuticals.

### 6.3 Future perspectives

The evolution of CAR T-cell therapy is characterised by a decisive shift from blunt, irreversible “kill switches” toward sophisticated, clinically actionable control systems. In the near term (2–3 years), modular adaptor platforms (UniCAR, SUPRA CAR) and logic-gated receptors (synNotch, Tmod) will continue to generate pivotal clinical data, with expansion into solid tumours and combination with other modalities. The medium term (3–5 years) will likely see integration of multiple control layers—for example, modular adaptor control combined with NOT-gate logic for AML.

For solid tumours, the most promising future strategies combine armouring to counteract the immunosuppressive tumour microenvironment with multi-antigen targeting to address tumour heterogeneity and prevent antigen escape. Armoured CARs (TRUCKs) engineered to secrete immunomodulatory payloads—such as IL-7, IL-12, IL-18, or CCL19—or to express dominant-negative receptors (e.g., dnTGFβRII) have already demonstrated clinical proof-of-concept in hepatocellular carcinoma and lymphoma. The emerging paradigm for CAR-T therapy in solid tumours will likely involve the integration of multiple complementary engineering strategies. Microenvironmental remodelling via armouring (e.g., cytokine secretion, dominant-negative receptors) will be combined with modular adaptor platforms to enable flexible multi-antigen targeting without re-engineering the T-cell product, and with logic-gated receptors to restrict activation to tumours expressing defined antigen combinations. This integrated, multi-pronged approach—addressing immunosuppression, antigen heterogeneity, and off-tumour toxicity simultaneously—represents the most promising pathway to overcome the limitations of single-modality CAR-T therapies in solid malignancies.

The long term (10+ years) may bring physical control systems like optogenetics and sonogenetics to the clinic, provided current translational hurdles (vector size, immunogenicity, delivery hardware, exogenous chromophores) are overcome.

Critically, no single platform fits all cases; instead, the optimal approach must be guided by tumour biology and the clinical scenario. By matching controllable architectures to specific disease contexts, clinicians will gain the ability to dictate not only which antigen to target, but also precisely when, where, and how intensely the engineered immune response should be triggered. This paradigm shift will transform CAR-T cell therapy from a high-risk intervention into a flexible, well-controlled, and routine part of oncology practice.

## Glossary

AE: Adverse Event
ALL: Acute Lymphoblastic Leukaemia
AML: Acute Myeloid Leukaemia
CAR: Chimeric Antigen Receptor
CBER: Centre for Biologics Evaluation and Research
CDER: Centre for Drug Evaluation and Research
CDRH: Centre for Devices and Radiological Health
CEA: Carcinoembryonic Antigen
CR: Complete Response
CRi: Complete Response with Incomplete Blood Count Recovery
CRC: Colorectal Cancer
CRS: Cytokine Release Syndrome
EGFR: Epidermal Growth Factor Receptor
FDA: Food and Drug Administration (USA)
FUS: Focused Ultrasound
GI: Gastrointestinal
GMP: Good Manufacturing Practice
HLA: Human Leukocyte Antigen
HSC: Haematopoietic Stem Cell
HSP70: Heat Shock Protein 70
iCAR: Inhibitory Chimeric Antigen Receptor
iCasp9: Inducible Caspase 9
ITAM: Immunoreceptor Tyrosine-based Activation Motif
ITIM: Immunoreceptor Tyrosine-based Inhibitory Motif
LOH: Loss of Heterozygosity
MAS: Macrophage Activation Syndrome
MRD: Minimal Residual Disease
MSLN: Mesothelin
NCT: National Clinical Trial (identifier)
NGS: Next-Generation Sequencing
ORR: Overall Response Rate
PD-1: Programmed Death-1
PDAC: Pancreatic Ductal Adenocarcinoma
PSMA: Prostate-Specific Membrane Antigen
R/R: Relapsed/Refractory
RP2D: Recommended Phase 2 Dose
SAE: Serious Adverse Event
scFv: Single-chain Variable Fragment
TCR: T-Cell Receptor
TM: Targeting Module
TME: Tumour Microenvironment
Tmod: Logic-gated CAR T platform (A2 Biotherapeutics)
TRUCKs: T cells Redirected for Universal Cytokine Killing
UCNP: Upconversion Nanoparticle
